# What Should Be Considered in the Evidence-Based Practice Competency-Based Curriculum for Undergraduate Nursing Students? From the Student’s Point of View

**DOI:** 10.3390/ijerph182010965

**Published:** 2021-10-19

**Authors:** Chieun Song, Weongyeong Kim, Jeongmin Park

**Affiliations:** Department of Nursing, Nambu University, Gwangju 62271, Korea; s9583015@gmail.com (C.S.); mini0321@nambu.ac.kr (J.P.)

**Keywords:** evidence-based practice, nursing education, undergraduate, qualitative content analysis

## Abstract

Background: The nursing curriculum should be systematically developed to improve the evidence-based practice (EBP) competencies of undergraduate nursing students. We attempted to identify the factors important for developing or improving the EBP-integrated nursing curriculum. Methods: This study adopted the qualitative research design using qualitative content analysis. A total of 168 study participants were included in the study. The participants were third-year nursing students at a private university located in southeastern South Korea. An open-ended question was asked: “To improve students’ EBP competencies during theory classes, on-campus practicums, or clinical practicums, what do you think is necessary?” Result(s): The analysis presented thirty themes, which were grouped into 10 sub-categories and further into four categories, and finally into three main categories. The students responded that they needed to form their own attitudes toward EBP. Regarding educator-related needs, students responded that effective teaching and learning methods should be used in classes. The students also suggested that the EBP process should be applied during clinical practicum. Regarding school-related needs, students suggested that EBP education should be applied at the beginning of the curriculum. Furthermore, the students recommended that repeated teaching should be used for EBP, and that EBP education should be connected to the major courses. Conclusions: The development of EBP competencies among undergraduate students is an important factor that can impact the nursing quality and patient safety. Based on the findings of this study, multidimensional efforts are needed to improve the liberal arts education of students and strengthen the educators’ competencies of EBNP and EBTP. Furthermore, schools should strive to assess students’ educational needs regularly and integrate the subsequent EBP-integrated nursing curricula consistent with these needs.

## 1. Introduction

In recent years, the reform of health professions education to enhance quality and patient safety in the clinical setting has been emphasized [1]. As such, reforms have been actively underway in the field of nursing education. One of the five competencies of healthcare professionals is evidence-based practice (EBP) [1]. This competency is required not only at the advanced level, but also at the entry level of nursing education. The American Association of Colleges of Nursing (AACN) emphasizes the need for nurses to be knowledgeable in EBP and attain a level of competence to ensure confidence in the evidence when making decisions related to patient care [2]. Accordingly, active improvements have been made in undergraduate nursing education curricula [3,4].

EBP is a problem-solving approach for appropriate clinical decision-making and consists of three components: best evidence, clinical expertise, and the students’ values and preferences [5]. If evidence-based nursing practice (EBNP) is performed by nurses to improve the quality of nursing in clinical settings, evidence-based teaching practice (EBTP) must be facilitated by educators to improve the quality of nursing education [6]. An educator must have the ability to select well-designed education strategies to achieve learning outcomes based on best evidence [7] and have the expertise to implement them. In addition, education should reflect the values and learning preferences of students.

Previous research studies related to EBP among undergraduate students have quantitatively or qualitatively examined students’ EBP competencies [8,9] or reported on the students’ beliefs, self-efficacy, knowledge, or attitude towards EBP [10,11,12,13,14]. Furthermore, most of the literature reported on the effects of EBP courses or programs provided to undergraduate students [15,16,17,18]. Recent qualitative studies on students’ EBP experiences, used clinical practicum [8] or extracurricular activities, such as clinical projects [19], to explored students’ EBP competency within clinical settings. Existing literature lacks comprehensive surveys on students’ perspectives regarding their desired education (theory classes, on-campus practicums, or clinical practicums) to foster their EBP competency. With the current emphasis on the development of students’ EBP competencies within the undergraduate curriculum, an exploration of students’ values and needs regarding EBP education is essential for developing and improving EBP-based curricula.

Thus, this study aimed to explore the needs of undergraduate students who have completed an EBP-integrated research course in developing their EBP competencies. In analyzing those needs, we identified factors that should be considered when developing and improving curricula.

## 2. Materials and Methods

### 2.1. Research Design

This study employed a qualitative design, aiming to explore undergraduate nursing students’ needs for theoretical education, on-campus practicum, and clinical practicum education to foster their EBP competencies. Data collected were analyzed using qualitative content analysis.

### 2.2. Study Participants

The participants were 168 third-year undergraduate nursing students in a private university in G city, Southeastern South Korea, who had registered for a compulsory “Research Methodology” course. The university in question is a private, four-year university, in which the study participants had completed the nursing processes and Fundamental nursing courses in their second year and began their clinical practicum in the topics of adult nursing, pediatric nursing, women’s health nursing, and psychiatric nursing from the first semester of their third year. Within the current university curriculum, there is no regular, independent course on the theory or practice of EBP, along with the absence of core courses on nursing informatics or statistics. A 2 h long, 2-credit compulsory research methodology course is offered in the second semester of the third year for 15 weeks, structured to enable students to recognize the need for nursing research and EBP, and acquire relevant competencies. To explore the vivid experiences and needs of students who were close to completing the EBP-integrated research methodology course, only third-year students were included in this study. First- and second-year students were excluded as they had yet to learn the basics of EBP. Although fourth-year students had completed the EBP-integrated research methodology course in the previous year [14], they were excluded because the survey period was deemed inappropriate, considering it coincided with employment or preparation for the national licensing examination.

### 2.3. Data Collection Method

To analyze students’ needs, an open question was posed in week 15 of the 2020 research methodology course through the University Learning Management System (LMS): “To improve students’ competencies in EBP through theory, on-campus practicums or clinical practicums, what do you think is needed?” Dr. Song, the lead researcher—who is experienced in teaching an EBP-integrated research methodology course [14]—developed this open-ended question after recognizing the lack of a needs assessment as a quality improvement (QI) activity at the end of the semester. The developed item was reviewed and finalized by two nursing educators, who have taught an undergraduate research methodology course and are currently teaching theory and clinical practicum courses.

Data were collected during the last week of the course in adherence to the Declaration of Helsinki principles. Students were informed in advance regarding answering an open-ended question inquiring their EBP-related educational needs via the LMS, and they were provided with detailed instructions for participating in the survey. Students were also informed that their participation was voluntary and their response to the open-ended question would not affect their grades; moreover, they were informed of their right to withdraw at any time. The response data were downloaded as an Excel file from the LMS by the lead researcher, after which personally identifiable information was deleted, serial numbers were assigned, and they were converted into nonidentifiable data. Furthermore, students were informed that the response data would not be used for any purpose other than for this study, with special consideration to confidentiality.

Of the 168 students who had enrolled in the research methodology course, 159 (94.6%) responded. Among them, data of 9 students that did not contain quality responses were excluded. The data of 150 students (94.3%) were included in the final analysis.

### 2.4. Data Analysis

Inductive qualitative content analysis was conducted manually using the three steps (preparation, organization, and reporting of results) suggested by Elo and Kyngäs [20]. In the preparation stage, analysis units were determined by meaningful sentences or phrases. First, Dr. Song focused on and reviewed the entire data repeatedly with the study purpose in mind. Then, words or phrases indicating key thoughts or concepts were highlighted to extract meaning units. These meaning units were classified into 242 codes. In the organizing stage, the codified data were clustered to obtain broad themes, while striving to maintain objectivity in data interpretation. Two other researchers (Dr. Kim and Dr. Park) independently reviewed the appropriateness of the themes. The researchers verified the clarity and consistency of the extracted themes clearly with participants’ intentions and the raw data. Three researchers brainstormed together for this abstraction process. Subsequently, thirty themes emerged, and they were classified into 10 sub-categories, which were named and grouped in 4 categories and further into 3 main categories. During the organizing stage, any disagreements among the researchers were resolved through discussion. The completed result of organizing themes is presented in Table 1.

### 2.5. Trustworthiness

To ensure trustworthiness of the current study results, we used the checklist provided by Elo et al. [20]. In the preparation stage, we selected third-year students, who took a theoretical education course, clinical practicum, and EBP-integrated research methodology course, as the study participants to acquire representative responses for the study’s open-ended question. Further, we ensured an adequate sample size and responses for examining diverse needs. The unit of analysis was chosen such that it is not too narrow or broad, and we tried to fully describe the meaning unit in the reporting of results.

In the organizing stage, we provided a detailed explanation regarding theme identification and categorization, and we developed a moderate number of categories by excluding any redundant categories. Three researchers participated in the categorization process to verify accuracy and consistency of the developed categories with the raw data, and any disagreements were resolved by a discussion until a consensus was reached. These categorizations were also reviewed and reconfirmed by a nursing professor with strong experience in qualitative research.

In the reporting stage, we systematically and logically reported the results and used participants’ statements as samples to clearly convey their meaning. We reported the participant details and content analysis findings to facilitate reader clarity regarding the generalizability of the study findings.

## 3. Results

The results of the analysis of the open-ended question on the requirements to improve nursing students’ EBP competencies in undergraduate nursing education were classified into 10 sub-categories, 4 categories, and 3 main categories (student-related, educator-related, and school-related) (Table 1).

### 3.1. Student Related

#### 3.1.1. Forming an Attitude toward EBP

Participants responded that attitudes in the affective, behavioral, and cognitive domains were needed to develop EBP competencies. Affective components of attitude included having an interest in patients (caring) and the cultivation of a spirit of inquiry on the clinical situation or nursing intervention (inquiring). Other factors included recognizing the importance of EBP, having a positive attitude, and understanding the value of nursing (valuing), as well as having the willpower to implement EBP (willing to implement). In terms of behavioral attitude, the participants responded that active problem-solving, collaboration, and communication skills were necessary. Further, students believed that they must be accustomed to reading liberal arts literature before attempting to read difficult research articles. In terms of cognitive attitude, the participants responded that students should become accustomed to critical and creative thinking.

*“In order to improve students’ EBP competencies, I believe that having an interest towards patients is key”*.

(p. 41)

“*Since students are also prospective nurses, it is important that they get into the habit of asking questions and having question marks”*.

(p. 61)

“*I am confident that understanding and being aware of the purpose and significance of the concept of evidence-based nursing will lead to positive values and that the profession of nursing has potential to change practice*”.

(p. 82)

“*Above all, I believe that an individual’s strong will to make an effort is essential. Although it is common to be forced by external pressure, I believe that EBP competencies can be improved when there is a strong desire to do so*”.

(p. 35)

“*The ability to think critically and use the information needed to actively seek solutions to problems, as well as effective communication skills to solve problems with team members is necessary*”.

(p. 28)

“*Nowadays, students rarely read books. Although some may be due to being busy with their studies, many students may also feel repulsed by reading simply because they are not accustomed to reading. As asking these students to read journal articles may seem more difficult, starting by reading literature in their field of choice and reducing the burden and repulsion to ‘reading’ will enable them to find better quality evidence*”.

(p. 25)

“*The various clinical situations that can be observed in clinical practicums require creative and critical thinking…”*.

(p. 21)

#### 3.1.2. Developing Competencies for the Components of EBP

The participants responded that students should develop competencies required to obtain the best available evidence using clinical expertise while reflecting on patient values and preferences, and all the components of EBP. They responded that students should have a self-directed attitude for learning and acquire core knowledge of nursing and clinical skills to develop clinical expertise. Furthermore, students should become accustomed to asking clinical questions, search for the best evidence, develop their ability to search for evidence, and critically evaluate selected literature. To understand the values and preferences of patients, students should first have an interest in the clinical situation and the patients, and then develop their therapeutic communication and interpersonal skills.

“*One must acquire clinical expertise through accurate nursing knowledge and clinical skills obtained through direct experience, observation, and practice*”.

(p. 126)

“*I believe that having the ability to effectively find evidence and evaluate how reliable it is essential to providing quality care*”.

(p. 107)

“*One must become accustomed to asking clinical questions and share opinions regarding clinical questions framed in the PICO format during clinical practice*”.

(p. 160)

“*The reasons why nurses were unable to apply findings from nursing research to practice were lack of time, challenges in reviewing the literature, a lack of ability to evaluate the quality of research, lack of critical thinking skills, and working environment. I thought the reason students who later become nurses were not able to apply EBP would be similar to above, but since the lack of time or working environment are not an issue we can solve right away, we should be able to improve in other avenues, such as our ability to evaluate the quality of evidence, giving critique, and critical thinking skills*”.

(p. 14)

“*Patients’ values and preferences must be taken into consideration for EBP, and in order to pay greater attention to patients’ values and preferences, good communication with patients is essential*”.

(p. 58)

### 3.2. Educator Related

#### Effective Teaching and Learning Methods

The participants indicated that effective teaching and learning methods should be adopted in the classroom to improve EBP competencies. They stated the need to attract students’ interest and enable active interaction between students, and between students and the educator in theory-based courses, along with applying teaching methods such as discussions or Havruta learning. Furthermore, participants believed that providing assignments that involve the application of the EBP process would be helpful. In practical training, the participants demanded a clinical environment in which they could experience EBP. In particular, they suggested a clinical setting in which they could apply the EBP process, include EBP in their practicum logs and case studies, and experience the EBP process with a clinical nurse.

“*Demonstrating medical content (i.e., surgery, nursing, conflicts with patients, disease, etc.) in movies or shows and applying them as cases may attract students’ interests and encourage them to actively participate in class, developing critical thinking and problem-solving skills*.”.

(p. 158)

“*I believe that students’ questions and positive feedback from instructors are crucial for improving students’ EBP competencies*”.

(p. 84)

“*I believe that creating a clinical question on curiosities and finding and discussing relevant data can help improve EBP competencies*”.

(p. 154)

“*Engaging in several assignments that require creating a clinical question and solving it with a critical perspective will be greatly helpful in improving EBP competencies*”.

(p. 60)

“*Creating a clinical question during an actual clinical practicum and doing a case study to promote practical application. Creating a clinical question by oneself, finding and applying evidence, and learning practical skills while receiving feedback and supplementing weaknesses*”.

(p. 3)

“*If there is space for a clinical question in the practicum log as a means to delve into curiosities, even in menial things, it may be of great help in improving EBP competencies*”.

(p. 70)

### 3.3. School Related

#### School Culture and the Readiness for EBP

The participants also requested the improvement of curricula and noncompulsory programs for the improvement of EBP competencies. They suggested implementing EBP training from the first year, especially education for improving the use of search databases and literature comprehension. They proposed the need for extensive education and an EBP course on research methodology, along with repeated instructions to ensure that students become familiarized with EBP. The participants’ responses also suggested a need to connect EBP with compulsory courses, and between theory- and practice-based courses. They proposed implementing special lectures, academic conferences, and evidence-gathering competitions, as well as extracurricular activities, as a means to regularly expose students to EBP.

“*What if this course was applied in first-year? With content that can be fully understood by a first-year student…”*.

(p. 18)

“*I believe that education on DB should start in first year and that time to explore and make comparisons within one’s interests and come to realize which DB has more data is needed. Although it would be great if this could be done individually, if not, DB evidence-gathering competitions to enable greater exposure would also be great*”.

(p. 25)

“*I found it challenging to fully understand the contents discussed in literature through the 2-credit course. As this course was offered during a busy semester, it seemed even more challenging. Since there is a limit to course credits, if students can become accustomed to the literature through expert seminars or other programs on reading articles may enable students to more effectively use evidence during assignments and case studies*”.

(p. 137)

“*As EBP competencies cannot be strengthened through short-term training, it is best to implement components or skills of EBP early on in the nursing curriculum, and, as was done in this school, to apply contents related to EBP in existing topics starting from second year, when practicums start, or the first semester of third year. I believe that it is necessary to find a way to apply the knowledge and skills acquired in EBP courses by working with nurses through the clinical practicum*.”.

(p. 51)

“*Rather than seeming to challenge, a curriculum in which the concepts and general knowledge on EBP are taught in a theory course and clinical questions relevant to the clinical situations observed by students during clinical practicum are posed, along with searches for evidence, may help students obtain a more detailed understanding of the nursing environment and practice*”.

(p. 38)

## 4. Discussion

This study aimed to identify students’ EBP-related educational needs. Based on a qualitative content analysis of undergraduate nursing students’ thoughts on EBP training, it was possible to identify the competencies the students believed should be cultivated, and those that they wish the educators or schools to engage in. As mentioned in the introduction, previous studies examined students’ EBP competency levels, evaluated the program effects, or conducted interviews about EBP competencies while focusing on clinical practicum, with paucity of studies having a similar aim with our study. Thus, we discussed students’ needs pertinent to four aspects by analyzing the collected data.

First, it is necessary to develop stronger liberal arts education to cultivate a spirit of inquiry, critical thinking, communication, teamwork_collaboration, and reading skill among nursing students. The ultimate purpose of EBP is to facilitate appropriate clinical decision-making in clinical nursing situations [5]. The basic competencies in implementing EBP are having an interest and curiosity towards patients and clinical situations, and having critical thinking skills, along with communication and collaboration skills within the nursing team to enable the accurate identification of problems and active problem solving [5,21]. To obtain the best available evidence, the ability to understand literature and evaluate its quality is critical [22]. Nevertheless, the process of reading literature and evaluating the quality of evidence can foster a notion that “EBP is challenging”, leading to a negative attitude towards EBP amongst undergraduate students [14]. As such, reading skill and information literacy must be cultivated prior to reading nursing literature. The participants were self-aware that basic competencies must first be developed in order to acquire EBP competencies. To support this, a systematic revamp of the liberal arts education in the department of nursing is imperative. The essentials [2] of the AACN also emphasized the value of liberal education as one of the core competencies of professional nursing education. Moreover, the findings of this study support the works of Kooken et al., (2018), who integrated the humanities into the nursing curriculum [23], and McKie (2012), who reported that liberal nursing education involving the arts and humanities could cultivate graduate attributes among nursing students [24].

Second, active support for educators is needed to strengthen EBP educators’ competencies. The participants of this study demanded the effective use of teaching methods that considered students’ interest, motivation, and interactions when designing and teaching classes, along with the integration of EBP, not only in research methodology courses, but also in core courses. Given that organizational culture is a major factor of EBP implementation in education, champions play an important role in culture formation [5]. The educator will likely have to play this role within school organizations. Thus, educators should be informed of learner-centered innovative teaching methods that reflect the learning styles and preferences of students [25,26,27], and offer support through teaching delivery media, both online and offline [28,29]. Above all, a regular EBP competency-building program for educators may need to be implemented to foster positive attitude, knowledge, and skills pertaining to EBP training among other members of the staff [30,31]. Moreover, the operation of a mentoring program between educators who are experienced in EBP training and those who are new to the concept is likely to be helpful [32,33].

Third, improvement of the clinical practicum is needed for undergraduate students to experience the process of applying EBP in clinical practice. Students wished to experience EBP with a nurse in place of the traditional observatory clinical practicum and stated in their responses that using a case study or practicum log would be helpful. Blackman and Giles (2017) suggested the need for an integrated clinically focused EBP curriculum and reported that a substantial number of students were not gaining experience in applying EBP in a clinical practice environment, while undergraduate students who had experience with EBP had a higher sense of confidence than those who did not [9]. Malik, McKenna, and Plummer (2016) stated that a lack of hospital culture, in cases where clinical nurses were not adopters of EBP or failed to serve as mentors, may act as a barrier [34]. Thus, improvements of the clinical practicum environment for undergraduate nursing students must be made systematically in partnership with the teaching hospitals [35], with special attention to strengthening clinical nurses’ EBP competencies through active cooperation between the hospital and university, considering that the knowledge, skills, and attitudes of nurses who directly partake in the education of students can be a great influence in student learning [36].

Fourth, widespread effort is needed to improve the curriculum at the institutional and departmental levels to facilitate regular theory–practice EBP training sessions. The study participants suggested the need to expand theoretical education on EBP and nursing research and demanded that education on database searches and literature comprehension be provided in the earlier years, as well as theory- and practice-based EBP training and noncompulsory EBP courses. According to a study that analyzed EBP courses offered in Korea [37], although most universities were offering a nursing research course, very few offered an independent course on EBP. Efforts to find ways to efficiently improve EBP training within a given educational environment are needed, such as incorporating EBP into research methodology courses [14], using simulation [38,39], or developing and offering an EBP education program [18]. Furthermore, active support to facilitate the achievement of learning goals for each year of study is needed, in the form of connecting courses to fit the curriculum [3], a curriculum that ties theory and practice [40], or extracurricular activities such as journal clubs [41]. However, such changes cannot be achieved by one educator, but rather require the development of a comprehensive cooperative system between nursing department and the school administration to form an organizational culture that embraces EBP.

This study has some limitations.

First, this study employed only one open-ended question about EBP-related educational needs and analyzed the responses of third-year students. Therefore, there is a possibility that students’ educational needs for the overall curriculum were not adequately examined. Thus, future studies need to perform an in-depth analysis of the entire student body’s needs, using open-ended questions about various aspects such as the school’s EBP organizational culture and clinical practicum environment. Second, this study analyzed the responses to an open-ended question via the LMS; however, we did not utilize any qualitative research reporting checklists. A checklist such as the consolidated criteria for reporting qualitative research (COREQ) [42] should be utilized to ensure systematic and higher-quality results. Third, since this study is minimal risk research that analyzed data collected during a regular course, it was not subject to an ethical review by an Institutional Review Board. However, we provided a detailed explanation about the study procedure and methodology through an online channel and informed students about the freedom to participate or withdraw, in addition to explaining to students that their response to the open-ended question will not affect their grades. Fourth, this study analyzed nursing students’ needs in a single university; thus, these results need to be generalized with caution.

Despite these limitations, this study has significant implications as it identified the specific factors that need to be considered in EBP education from nursing students’ perspective. Our findings particularly confirmed that students want to obtain the EBP process experience alongside nurses during their clinical practicum, participate in various extracurricular activities to promote their EBP competencies, and familiarize themselves with EBP through repetitive EBP education. Such findings may be used as preliminary data by educators or school administrators when developing and improving their curricula. Further research needs to explore the educational experiences of graduates, as well as current students, in depth to implement systematic and effective improvements for the EBP curriculum.

## 5. Conclusions

The development of EBP competencies among undergraduate students is an important factor that can impact the quality of nursing care and patient safety. Thus, it must be facilitated systematically in undergraduate nursing education. Just as EBP is emphasized in clinical practice, evidence-based education must be provided to learners, along with a reflection of students’ values and preferences. Based on the findings of this study, efforts at the institutional level, along with efforts made by educators, are needed to improve the liberal arts education of students and strengthen the educators’ competencies of EBNP and EBTP. Furthermore, academic–hospital partnerships must be established for the quality improvement of clinical practice education, along with multidimensional efforts to incorporate EBP education into all the years of study and corresponding courses.

## Figures and Tables

**Table 1:** Results of qualitative analysis.

Main Category	Category	Sub-Category	Theme
Student-related	Attitude toward EBP	Affective domain-related	Caring
			Inquiring
			Valuing
			Willing to implement
		Behavior domain-related	Active problem solving
			Teamwork_Collaboration
			Communication
			Reading skill
		Cognitive domain-related	Critical thinking
			Creative thinking
	Components of EBP	Clinical expertise-related	Nursing knowledge
			Clinical competency
		Best available evidence-related	Formulating clinical question
			Searching for the best evidence
			Critically appraising the evidence
		Patient values and preferences-related	Therapeutic communication skills
			Interpersonal skills
Educator-related	Teaching and learning method	Classroom-related	Strategies to develop students’ interests
			Interaction (S–S, S–T)
			Effective instructional strategies
			Assignment
		Practicum-related	Providing an environment where EBP can be applied
School-related	School culture for the readiness of EBP	Curriculum-related	Early introduction of EBP into curriculum
			EBP course opening
			Repeated education
			Linked courses operation
		Extra curriculum-related	Special lectures
			Academic seminar
			Club activity
			Evidence searching competition or program

EBP = evidence-based practice; S–S = student–student; S–T = student–teacher.

## Data Availability

The data presented in this study are available on request from the corresponding author. The data are not publicly available due to privacy.

## References

[1] Institute of Medicine Committee on the Health Professions Education Summit (2003). Health Profession Education: A Bridge to Quality.

[2] American Association of Colleges of Nursing (2021). The Essentials: Core Competencies for Professional Nursing Education.

[3] Hung H.Y., Wang Y.-W., Feng J.-Y., Wang C.-J., Lin E.C.-L., Chang Y.-J. (2019). Evidence-based practice curriculum development for undergraduate nursing students: The preliminary results of an action research study in Taiwan. J. Nurs. Res..

[4] Disler R.T., White H., Franklin N., Armari E., Jackson D. (2019). Reframing evidence-based practice curricula to facilitate engagement in nursing students. Nurse Educ. Pract..

[5] Melnyk B.M., Fineout-Overholt E. (2019). Evidence-Based Practice in Nursing & Healthcare: A Guide to Best Practice.

[6] Kalb K.A., O’conner-Von S.K., Brockway C., Rierson C.L., Sendelbach S. (2015). Evidence-based teaching practice in nursing education: Faculty perspectives and practices. Nurs. Educ. Perspect..

[7] Diery A., Vogel F., Knogler M., Seidel T. (2020). Evidence-based practice in higher education: Teacher educators’ attitudes, challenges, and uses. Frontiers in Education. Front. Educ..

[8] Lam C.K., Schubert C. (2019). Evidence-based practice competence in nursing students: An exploratory study with important implications for educators. Worldviews Evid. Based Nurs..

[9] Blackman I.R., Giles T. (2017). Can nursing students practice what is preached? Factors impacting graduating nurses’ abilities and achievement to apply evidence-based practices. Worldviews Evid. Based Nurs..

[10] Cardoso D., Rodrigues M., Pereira R., Parola V., Coelho A., Ferraz L., Cardoso M.L., Ramis M.-A., Apóstolo J. (2021). Nursing educators’ and undergraduate nursing students’ beliefs and perceptions on evidence-based practice, evidence implementation, organizational readiness and culture: An exploratory cross-sectional study. Nurse Educ. Pract..

[11] Amit-Aharon A., Melnikov S., Warshawski S. (2020). The effect of evidence-based practice perception, information literacy self-efficacy, and academic motivation on nursing students’ future implementation of evidence-based practice. J. Prof. Nurs..

[12] Laske R.A., Kurz J. (2019). Examining evidence-based practice beliefs in undergraduate nursing students: A pilot study. Teach. Learn. Nurs..

[13] Sánchez-García I., Molina M.D.P.U., López-Medina I.M., Pancorbo-Hidalgo P.L. (2019). Knowledge, skills and attitudes related to evidence-based practice among undergraduate nursing students: A survey at three universities in Colombia, Chile and Spain. Nurse Educ. Pract..

[14] Song C.E., Park H., Lee M., Stevens K.R. (2021). Integrating EBP into an undergraduate research methodology course using the Star Model of Knowledge Transformation: A mixed-method study. Nurse Educ. Today.

[15] Cosme S., Milner K.A., Wonder A. (2018). Benchmarking of prelicensure nursing students’ evidence-based practice knowledge. Nurse Educ..

[16] Reid J., Briggs J., Carlisle S., Scott D., Lewis C. (2017). Enhancing utility and understanding of evidence based practice through undergraduate nurse education. BMC Nurs..

[17] Kim J.S., Gu M.O., Chang H. (2019). Effects of an evidence-based practice education program using multifaceted interventions: A quasi-experimental study with undergraduate nursing students. BMC Med. Educ..

[18] Oh E.G., Yang Y.L. (2019). Evidence-based nursing education for undergraduate students: A preliminary experimental study. Nurse Educ. Pract..

[19] André B., Aune A.G., Brænd J.A. (2016). Embedding evidence-based practice among nursing undergraduates: Results from a pilot study. Nurse Educ. Pract..

[20] Elo S., Kääriäinen M., Kanste O.I., Pölkki T., Utriainen K., Kyngäs H. (2014). Qualitative content analysis: A focus on trustworthiness. SAGE Open.

[21] Dang D., Dearholt S.L. (2018). Johns Hopkins Nursing Evidence-Based Practice: Model and Guidelines.

[22] Howard V. (2021). Undergraduate mental health nursing students’ reflections in gaining understanding and skills in the critical appraisal of research papers—An exploration of barriers and enablers. Nurse Educ. Pract..

[23] Kooken W.C., Kerr N. (2018). Blending the liberal arts and nursing: Creating a portrait for the 21st century. J. Prof. Nurs..

[24] McKie A. (2012). Using the arts and humanities to promote a liberal nursing education: Strengths and weaknesses. Nurse Educ. Today.

[25] Milner K.A., McCloud R., Cullen J. (2020). The Evidence appraisal game: An innovative strategy for teaching step 3 in evidence-based practice. Worldviews Evid. Based Nurs..

[26] Gabriel P.M., Lieb C.L., Holland S., Ballinghoff J., Cacchione P.Z., McPeake L. (2021). Teaching evidence-based sepsis care: A sepsis escape room. J. Contin. Educ. Nurs..

[27] Horntvedt M.-E.T., Nordsteien A., Fermann T., Severinsson E. (2018). Strategies for teaching evidence-based practice in nursing education: A thematic literature review. BMC Med. Educ..

[28] Carrington J.M., Pace T.W.W., Sheppard K.G., Dudding K.M., Stratton D. (2017). Using twitter to teach evidence-based practice in doctor of nursing practice degree program. Clin. Nurse Spec..

[29] Ferguson C., DiGiacomo M., Gholizadeh L., Ferguson L.E., Hickman L.D. (2017). The integration and evaluation of a social-media facilitated journal club to enhance the student learning experience of evidence-based practice: A case study. Nurse Educ. Today.

[30] Lehane E., Leahy-Warren P., O’Riordan C., Savage E., Drennan J., O’Tuathaigh C., O’Connor M., Corrigan M., Burke F., Hayes M. (2019). Evidence-based practice education for healthcare professions: An expert view. BMJ Evid. Based Med..

[31] Aranas F.C.T. (2019). Effectiveness of evidence-based practice (EBP) training program on the level of competencies of faculty members in a school of nursing. Int. J. Nurs. Educ..

[32] Dahlke S., Raymond C., Penconek M.T., Swaboda B.N. (2021). An integrative review of mentoring novice faculty to teach. J. Nurs. Educ..

[33] Moore W.L. (2021). Does faculty experience count? A quantitative analysis of evidence-based testing practices in baccalaureate nursing education. Nurs. Educ. Perspect..

[34] Malik G., McKenna L., Plummer V. (2016). Facilitators and barriers to evidence-based practice: Perceptions of nurse educators, clinical coaches and nurse specialists from a descriptive study. Contemp. Nurse.

[35] Niederhauser V. (2016). Better Together: A win-win pediatric academic partnership. Pediatr. Nurs..

[36] Fiset V.J., Graham I.D., Davies B.L. (2017). Evidence-based practice in clinical nursing education: A scoping review. J. Nurs. Educ..

[37] Song C.E., Kim W.G., Lim Y.J. (2019). An analysis of evidence-based practice courses in Korean nursing education systems. Heliyon.

[38] Lewis K.A., Ricks T.N., Rowin A., Ndlovu C., Goldstein L., McElvogue C. (2019). Does simulation training for acute care nurses improve patient safety outcomes: A systematic review to inform evidence-based practice. Worldviews Evid. Based Nurs..

[39] Opsahl A., Nelson T., Madeira J., Wonder A.H. (2020). Evidence-based, ethical decision-making: Using simulation to teach the application of evidence and ethics in practice. Worldviews Evid. Based Nurs..

[40] Malik G., McKenna L., Griffiths D. (2017). Envisaging the use of evidence-based practice (EBP): How nurse academics facilitate EBP use in theory and practice across Australian undergraduate programmes. J. Clin. Nurs..

[41] Mayer D.D.M. (2019). Nursing journal club to teach EBP critical appraisal skills. Worldviews Evid. Based Nurs..

[42] Buus N., Perron A. (2020). The quality of quality criteria: Replicating the development of the consolidated criteria for reporting qualitative research (COREQ). Int. J. Nurs. Stud..

